# Features and Associated Factors of the Behavioral Development of 24-month-old Children in Rural China: Follow-up Evaluation of a Randomized Controlled Trial

**DOI:** 10.1038/s41598-018-32171-1

**Published:** 2018-09-18

**Authors:** Xue Yang, Zhaoyang Yin, Yue Cheng, Wenfang Yang, Zhonghai Zhu, Min Zhang, Danyang Li, Danli Liu, Hong Yan, Lingxia Zeng

**Affiliations:** 1 Department of Epidemiology & Biostatistics, School of Public Health, Xi’an Jiaotong University Health Science Center, Xi’an, Shaanxi Province 710061 China; 2 Department of Health, Northwest Women’s and Children’s Hospital, Xi’an, Shaanxi Province 710061 China; 3 Department of Pediatrics, the Shangluo Central Hospital, Shang Luo, Shaanxi Province 726000 China; 4 Department of Prevention and Health Care, the First Affiliated Hospital of Medical College in Xi’an Jiaotong University Health Science Center, Xi’an, Shaanxi Province 710061 China; 5 Nutrition and Food Safety Engineering Research Center of Shaanxi Province, Xi’an, Shaanxi Province 710061 China

## Abstract

The aim of this study was to assess the risk factors associated with the behavioral development among 24-month-old children in rural northwestern China. A total of 657 children whose mothers had participated in a double-blinded, randomized, controlled trial of antenatal micronutrient supplementation in western China were followed until 24 months of age. Their mental, psychomotor, and behavioral development were assessed by the Bayley Scales of Infant Development. Multivariate logistic regression models were used to examine the factors associated with infant behavioral development. Six behavioral factors of infants were presented: activity, social adaptability, reactivity, endurance, concentration, and motor coordination. Further analysis demonstrated that maternal malnutrition, exposure to risk factors during pregnancy, and adverse birth outcomes negatively affected the behavioral development of children at 24 months, which is a common co-occurrence with cognitive and emotional problems. These results suggest that strategies to improve infant behavioral development should consider the maternal pregnancy status.

## Introduction

The WHO estimates that nearly one in five children and adolescents encounter emotional or behavioral problems at some point in their life between childhood and adolescence^1^. Evidence suggests that children in China have more emotional and behavioral problems than children in some other developed countries, such as America and Germany^2,3^. In China, the prevalence of behavioral problems has reached 12.97% in adolescents^4^ and 15.63% in preschool children^5^. Extensive research has indicated that behavioral problems identified during the preschool years often persist moderately up to preadolescence and result in social maladaptation, emotional disorders, and mental disorders^6^, even leading to antisocial and illegal behavior in adulthood^7^.

Numerous factors influence the behavioral development of children, such as the conditions of the fetus in utero and/or at birth, household economics, and parental educational level. Adverse events and exposure during pregnancy, delivery, and the neonatal period have consistently been reported to be risk factors for cognitive delays and behavioral problems^8^. In particular, there is growing evidence that children born with a low birth weight, preterm, or small for gestational age may be prone to behavioral problems in adolescence^9,10^. A number of large-scale community-based trials have demonstrated that prenatal micronutrient supplementation was a cost-effective measure to prevent adverse birth outcomes, including low birth weight, preterm birth and small for gestation age, in middle-low income countries and contributed to improve the intellectual development of offspring^11,12^. However, the impact of prenatal micronutrient supplementation on the behavioral development of infants is not clear.

The first 1000 days of life is a critical period of development and may determine children’s health later in life^13^; meanwhile, from 2 to 6 years old is a key transition period when children move from the dependency of the preschool infant to the growing autonomy and social and cognitive competence of the school-aged child. Thus, it is important to differentiate between normal and abnormal behavioral development at 24 months of age^14^. Furthermore, behavioral problems may have adverse effects on social functioning and school performance, and assessment at an early age provides an opportunity to intervene and possibly prevent major problems in these areas^15^.

Therefore, we conducted this follow-up assessment of a population-based randomized controlled trial to analyze the features and associated factors of behavioral development among children at 24 months of age in rural northwestern China. We focused on the modifiable risk factors associated with infant behavioral development based on maternal nutrient status, perinatal conditions, birth outcomes, and early nutritional status of the infants to provide evidence for addressing the early intervention strategies of behavioral development in children in rural areas.

## Results

### Background characteristics of children and their households

**Figure 1:**
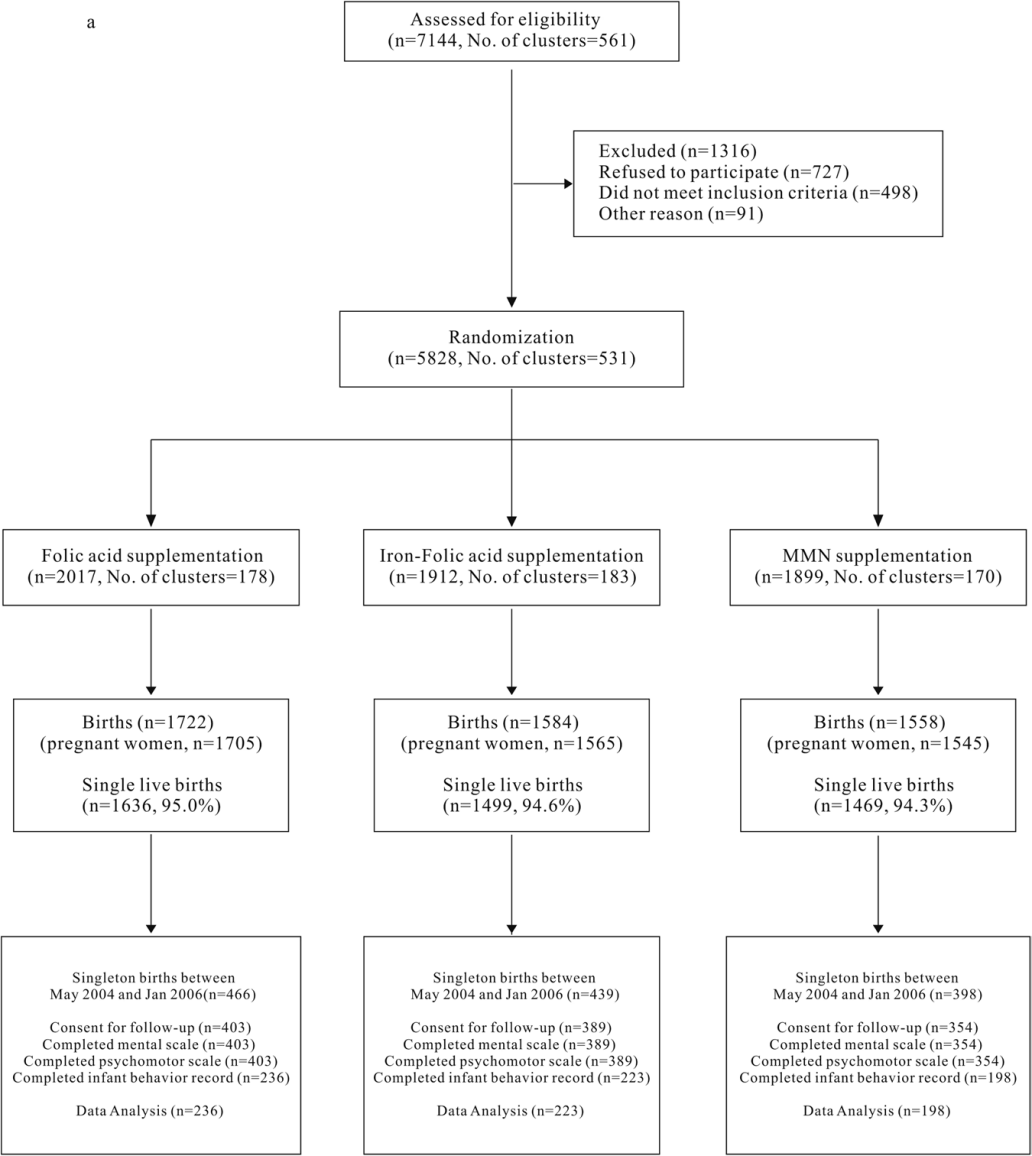
Participant flow chart.

The enrollment, allocation, and follow-up profiles are presented in Fig. 1. A total of 1303 of 4864 (26.8%) infants met the inclusion criteria and participated in the follow-up study. Mothers of 1146 infants agreed to the assessment at 24 months of age. In total, 657 infants completed the Bayley Scales of Infant Development (BSID-II) assessment.

Among the 657 infants, 404 (61.5%) were boys and 253 (38.5%) were girls (Table 1), with a mean gestational age at birth of 39.9 ± 1.6 weeks and a mean birth weight of 3179.5 ± 409.0 g. Grandparents were the primary caregivers of 46.7% of the infants. The occupations of most mothers (84.5%) and fathers (74.9%) were related to farming. Approximately 86.9% of the mothers and 78.4% of the fathers had only a primary or secondary school education.

**Table 1:** Background characteristics of the women and their offspring

Characteristic	N (%)/mean (SD)
Demographic characteristics	
Maternal education level, n (%)	
Primary school or less	175 (26.6)
Secondary school	396 (60.3)
High school or above	86 (13.1)
Maternal occupation, n (%)	
Farmer	555 (84.5)
Other	102 (15.5)
Paternal education level, n (%)^a^	
Primary school or less	65 (9.9)
Secondary school	449 (68.5)
High school or above	140 (21.4)
Paternal occupation, n (%)	
Farmer	492 (74.9)
Other	165 (25.1)
Household wealth during pregnancy, n (%)	
Poor	138 (21.0)
Middle	271 (41.3)
Wealthy	248 (37.8)
Maternal nutritional status and micronutrient supplements during pregnancy	
Age at delivery, mean ± SD, y	25.5 ± 4.6
MUAC in the first trimester, mean ± SD, cm^b^	23.1 ± 1.8
Micronutrient supplements, n (%)	
Folic acid	236 (35.9)
Iron-folic acid	223 (33.9)
MMN	198 (30.1)
No. of supplement tablets, mean ± SD, p	173.3 ± 41.1
Birth outcome	
Gestation age at birth, mean ± SD, w	39.9 ± 1.6
Birth weight, mean ± SD, g^c^	3179.5 ± 409.0
Birth length, mean ± SD, cm	49.2 ± 2.5
Head circumference, mean ± SD, cm	33.2 ± 1.6
Apgar score at 5 min after birth, mean ± SD	9.9 ± 0.5
Gender, n (%)	
Boy	404 (61.5)
Girl	253 (38.5)
Toddler characteristics at 24 months of age	
Age at the assessment, mean ± SD, d	24.5 ± 0.6
Length, mean ± SD, cm	84.6 ± 3.3
Weight, mean ± SD, kg	11.7 ± 1.3
Primary caregivers, n (%)^d^	
Parents	342 (53.3)
Grandparents	300 (46.7)

SD, standard deviation; MUAC, mid-upper arm circumference; MMN, multiple micronutrient. ^a^Paternal education level missing 3. ^b^MUAC missing 7. ^c^Birth weight missing 10. ^d^Primary caregivers missing 15.

Most of the background characteristics of the children who finished the BSID assessment and the children who did not were comparable. The mean Apgar scores of the infants at 5 minutes after birth, the mean age of the toddlers at assessment and the mean number of supplement tablets consumed were significantly different between the group that completed the BSID and the group that did not complete the BSID; however, the absolute differences were small (Additional file 1: Supplementary Table S1).

### Features of behavioral development of children at 24 months of age in rural northwest China

The inter-rater reliability of the BSID scores from 10 infants was evaluated by 2 specific examiners, and the result of the raters’ consistency was 0.86 (Kappa) (Chi-squared test, *P* < 0.001). The intra-class correlation coefficients were 0.99 for the mental scale, 0.99 for the psychomotor scale and 0.96 for the infant behavior record (IBR) scale in all subjects, indicating ‘excellent’ reliability.

Among mental and psychomotor scales, 24-month-old infants had a mean mental development index (MDI) score of 86.0 ± 17.2 and a mean psychomotor development index (PDI) score of 101.9 ± 13.3 (Table 2). For behavioral items, the higher scores were obtained for emotional tone (4.0 ± 0.5) and endurance behavior (4.0 ± 0.4), meanwhile, lower scores were obtained for fearfulness (1.8 ± 0.8) and sucking of thumb or fingers (1.2 ± 0.6).

**Table 2:** Record of BSID items in infants at 24 months of age.

BSID Items	mean ± SD	*M* (*Q*_25_, *Q*_75_)
MD raw score	142.2 ± 5.2	142.0 (139.0, 145.0)
PD raw score	62.8 ± 2.7	63.0 (61.0, 65.0)
MDI	86.0 ± 17.2	85.6 (75.9, 97.3)
PDI	101.9 ± 13.3	99.2 (92.2, 109.4)
Behavioral items		
Social orientation	11.1 ± 1.1	11.0 (10.0, 12.0)
To persons	3.7 ± 0.6	4.0 (3.0, 4.0)
To examiner	3.4 ± 0.6	3.0 (3.0, 4.0)
To mother	3.9 ± 0.4	4.0 (4.0, 4.0)
Cooperativeness	3.7 ± 0.8	4.0 (3.0, 4.0)
Fearfulness	1.8 ± 0.8	2.0 (1.0, 2.0)
Tension	2.5 ± 0.7	2.0 (2.0, 3.0)
Emotional tone	4.0 ± 0.5	4.0 (4.0, 4.0)
Object orientation	3.9 ± 0.4	4.0 (4.0, 4.0)
Goal directedness	3.1 ± 0.7	3.0 (3.0, 3.0)
Attention span	3.5 ± 0.6	4.0 (3.0, 4.0)
Endurance	4.0 ± 0.4	4.0 (4.0, 4.0)
Activity	3.5 ± 0.6	3.0 (3.0, 4.0)
Reactivity	3.7 ± 0.5	4.0 (3.0, 4.0)
Sensory areas of interest	27.2 ± 2.2	27.0 (26.0, 29.0)
Sight	3.8 ± 0.5	4.0 (4.0, 4.0)
Listening	2.7 ± 0.7	3.0 (2.0, 3.0)
Vocalizing	3.0 ± 0.7	3.0 (3.0, 3.0)
Banging	2.8 ± 0.6	3.0 (2.0, 3.0)
Manipulating	3.5 ± 0.6	4.0 (3.0, 4.0)
Body motion	3.4 ± 0.6	3.0 (3.0, 4.0)
Sucking thumb or fingers	1.2 ± 0.6	1.0 (1.0, 1.0)
Sucking pacifier	3.1 ± 0.9	3.0 (2.0, 4.0)
Sucking toys	3.7 ± 0.5	4.0 (3.0, 4.0)
Energy and coordination	9.1 ± 1.2	9.0 (8.0, 10.0)
Level	3.1 ± 0.5	3.0 (3.0, 3.0)
Gross muscle	3.0 ± 0.5	3.0 (3.0, 3.0)
Fine muscle	3.0 ± 0.7	3.0 (3.0, 3.0)

BSID, Bayley scale of infant development; SD, standard deviation; *M*, median; *Q*, quartile; MD, mental development; PD, psychomotor development; MDI, mental development index; PDI, psychomotor development index.

Factor analysis was conducted on the IBR scale of 25 item scores of the 657 infants. This analysis showed only two primary factors with eigenvalues of 3.932 and 3.053, which indicated a reasonably high level of reliability for the BSID scores. Six behavioral factors were extracted by factor analysis: activity, social adaptability, reactivity, endurance, concentration, and motor coordination (Table 3). These behavioral factors accounted for 55.46% of the variation. Of all the behavioral factors, activity and social adaptability had the highest percentages of total variance: 15.73% and 12.21%, respectively.

**Table 3:** Activity, social adaptability, reactivity, endurance, concentration, and motor coordination were extracted for the IBR at 24 months of age.

IBR Items		Factors					
No.	Name	I	II	III	IV	V	VI
20	Manipulating	0.83					
4	Cooperativeness	0.73					
24	Sucking toys	0.73					
21	Body motion	0.59					
25	Energy	0.56					
16	Sight	0.56					
17	Listening		0.75				
2	Social with examiner		0.72				
19	Banging		0.72				
5	Fearfulness		−0.56				
18	Vocalizing		0.54				
3	Social with mother		0.41				
27	Fine muscle			0.75			
1	Social with persons			0.61			
15	Reactivity			0.44			
7	Emotional tone				0.64		
6	Tension				−0.57		
13	Endurance				0.53		
11	Goal directedness					0.68	
14	Activity					−0.56	
23	Sucking pacifier					0.43	
12	Attention span					0.41	
26	Gross muscle						−0.65
22	Sucking thumb or fingers						−0.64
8	Object orientation						0.45
Percentage of total variance		15.73	12.21	7.31	7.17	6.59	6.45

IBR, infant behavior record. Factor I: activity; Factor II: social adaptability; Factor III: reactivity; Factor IV: endurance; Factor V: concentration; Factor VI: motor coordination.

### The factors associated with behavioral development in children at 24 months

With the exception of motor coordination, the other 5 behavioral factors correlated with the MDI, with correlation coefficients ranging from 0.103 to 0.354 (Spearman’s coefficient, *P* < 0.05) (Table 4). However, only reactivity and concentration correlated with the PDI with significance; the correlation coefficients were 0.139 and −0.145 (Spearman’s coefficient, *P* < 0.05), respectively. However, the partial correlation analysis with adjustments for relevant covariates showed a non-significant difference between reactivity and the MDI; the adjusted correlation coefficient was 0.044 (Partial coefficient, *P* = 0.274). Of the behavioral factors, activity showed the highest positive correlation with the MDI (Partial coefficient, r = 0.371, *P* < 0.001), and concentration showed a negative correlation with the PDI (Partial coefficient, r = −0.120, *P* = 0.003).

**Table 4:** Spearman coefficients and partial correlation coefficients between the IBR factor components and Bayley’s MDI and PDI at 24 months of age

Factors	MDI				PDI			
	*r*	*P*	r*	*P* ^*a*^	*r*	*P*	r*	*P* ^*a*^
Activity	0.354	<0.001	0.371	<0.001	0.054	0.170	0.034	0.397
Social adaptability	0.175	<0.001	0.153	<0.001	0.012	0.756	0.002	0.963
Reactivity	0.103	0.008	0.044	0.274	0.139	<0.001	0.107	0.008
Endurance	0.115	0.003	0.101	0.012	0.074	0.058	0.067	0.100
Concentration	0.134	0.001	0.203	<0.001	−0.145	<0.001	−0.120	0.003
Motor coordination	−0.030	0.446	0.018	0.660	0.063	0.110	0.065	0.110

IBR, infant behavior record; MDI, mental development index; PDI, psychomotor development index; r, correlation coefficient. *r and ^a^*P* values were adjusted according to maternal micronutrient supplementation (treatment), number of supplement tablets consumed, age of children at assessment, gestational week at delivery, infants’ birth weight, mother’s age at delivery, MUAC at delivery, maternal education level and occupation, paternal education level and occupation, household wealth, and primary caregivers of infant.

Among 657 women, 405 were malnourished, 18 were exposed to alcohol, and 368 were exposed to smoke during their pregnancy. Univariate analysis showed that maternal toxic chemical exposure, maternal alcohol exposure, infant malnutrition, and poor household wealth were related to decreased standardized scores of behavioral factors in activity (Additional file 1: Supplementary Table S2).

Similarly, multivariable logistic regression analysis showed that maternal alcohol exposure was significantly associated with abnormal activity level in infants [odds ratio (OR) = 0.28; 95% confidence interval (CI), 0.10–0.83]. Moreover, compared with the “maternal folic acid supplementation” subgroup, the “iron-folic acid” subgroup (OR = 0.91; 95% CI, 0.56–1.46) and the “multiple micronutrient” (MMN) subgroup (OR = 0.52; 95% CI, 0.31–0.89) showed results that were consistent with a decreased risk of infant social adaptability behavioral defects. Compared with the “poor household wealth” subgroup, the “middle” and “wealthy” subgroups were positively associated with infant social adaptability and endurance levels, with a magnitude of OR differences ranging from 0.40 to 0.70 (Table 5).

**Table 5:** Factors associated with infant behavioral development at 24 months of age

Index	N (%)	Activity		Social adaptability		Reactivity		Endurance		Concentration		Motor coordination	
		n (%)	OR (95% CI)	n (%)	OR (95% CI)	n (%)	OR (95% CI)	n (%)	OR (95% CI)	n (%)	OR (95% CI)	n (%)	OR (95% CI)
Maternal nutrition status and adverse exposure during pregnancy													
MUAC^a^													
≤23.5	405 (61.6)	85 (21.0)	1.00	84 (20.7)	1.00	82 (20.2)	1.00	85 (21.0)	1.00	78 (19.3)	1.00	85 (21.0)	1.00
>23.5	245 (37.3)	45 (18.4)	0.91 (0.60, 1.40)	46 (18.8)	0.92 (0.60, 1.43)	49 (20.0)	1.02 (0.67, 1.55)	46 (18.8)	0.85 (0.56, 1.31)	53 (21.6)	1.17 (0.77, 1.77)	45 (18.4)	0.79 (0.52, 1.21)
Micronutrient supplementation													
Folic acid	236 (35.9)	45 (19.1)	1.00	57 (24.2)	1.00	47 (19.9)	1.00	53 (22.5)	1.00	46 (19.5)	1.00	42 (17.8)	1.00
Iron –folic acid	223 (33.9)	53 (23.8)	1.31 (0.81, 2.12)	45 (20.2)	0.91 (0.56, 1.46)	49 (22.0)	0.93 (0.58, 1.50)	39 (17.5)	0.64 (0.39, 1.06)	45 (20.2)	1.00 (0.61, 1.63)	42 (18.8)	1.04 (0.63, 1.71)
MMN	198 (30.1)	33 (16.7)	0.85 (0.50, 1.42)	30 (15.2)	0.52 (0.31, 0.89)	35 (17.7)	0.73 (0.44, 1.21)	40 (20.2)	0.79 (0.49, 1.29)	40 (20.2)	1.05 (0.64, 1.72)	47 (23.7)	1.42 (0.87, 2.30)
No. of supplement tablets													
≤180	325 (49.5)	66 (20.3)	1.00	75 (23.1)	1.00	72 (22.2)	1.00	74 (22.8)	1.00	64 (19.7)	1.00	62 (19.1)	1.00
>180	332 (50.5)	65 (19.6)	0.95 (0.63, 1.42)	57 (17.2)	0.69 (0.46, 1.04)	59 (17.8)	0.81 (0.54, 1.21)	58 (17.5)	0.74 (0.49, 1.11)	67 (20.2)	1.06 (0.71, 1.59)	69 (20.8)	1.11 (0.74, 1.65)
Smoke exposure													
Yes	368 (56.0)	77 (20.9)	1.00	67 (18.2)	1.00	75 (20.4)	1.00	80 (21.7)	1.00	67 (18.2)	1.00	79 (21.5)	1.00
No	289 (44.0)	54 (18.7)	0.94 (0.62, 1.42)	65 (22.5)	1.51 (0.99, 2.30)	56 (19.4)	0.93 (0.61, 1.42)	52 (18.0)	0.74 (0.48, 1.13)	64 (22.2)	1.47 (0.97, 2.22)	52 (18.0)	0.78 (0.51, 1.18)
Toxic chemical exposure													
Yes	153 (23.3)	39 (25.5)	1.00	33 (21.6)	1.00	35 (22.9)	1.00	33 (21.6)	1.00	30 (19.6)	1.00	32 (20.9)	1.00
No	504 (76.7)	92 (18.3)	0.71 (0.45, 1.13)	99 (19.6)	1.06 (0.65, 1.75)	96 (19.1)	0.83 (0.52, 1.34)	99 (19.6)	1.02 (0.62, 1.66)	101 (20.0)	1.00 (0.61, 1.64)	99 (19.6)	0.92 (0.56, 1.47)
Alcohol exposure													
Yes	18 (2.7)	7 (38.9)	1.00	1 (5.6)	1.00	4 (22.2)	1.00	2 (11.1)	1.00	3 (16.7)	1.00	2 (11.1)	1.00
No	626 (95.3)	123 (19.7)	0.28 (0.10, 0.83)	126 (20.1)	4.83 (0.61, 38.48)	123 (19.7)	1.16 (0.31, 4.28)	127 (20.3)	3.99 (0.51, 31.48)	125 (20.0)	1.55 (0.40, 6.01)	126 (20.1)	1.37 (0.30, 6.32)
Household wealth during pregnancy													
Poor	138 (21.0)	24 (17.4)	1.00	41 (29.7)	1.00	35 (25.4)	1.00	36 (26.1)	1.00	26 (18.9)	1.00	32 (23.2)	1.00
Middle	271 (41.3)	66 (24.4)	1.72 (0.99, 2.99)	55 (20.3)	0.59 (0.35, 0.98)	52 (19.2)	0.77 (0.46, 1.30)	55 (20.3)	0.70 (0.42, 1.17)	56 (20.7)	1.30 (0.75, 2.28)	47 (17.3)	0.79 (0.46, 1.35)
Wealthy	248 (37.8)	41 (16.5)	1.18 (0.65, 2.15)	36 (14.5)	0.40 (0.22, 0.70)	44 (17.7)	0.80 (0.46, 1.39)	41 (16.5)	0.53 (0.30, 0.92)	49 (19.8)	1.19 (0.66, 2.14)	52 (21.0)	1.07 (0.62, 1.84)
Adverse birth outcomes													
LBW^b^													
Yes	26 (4.0)	6 (23.1)	1.00	5 (19.2)	1.00	6 (23.1)	1.00	7 (26.9)	1.00	11 (42.3)	1.00	4 (15.4)	1.00
No	621 (94.5)	123 (19.8)	0.68 (0.23, 2.01)	124 (20.0)	1.19 (0.35, 4.07)	123 (19.8)	1.06 (0.37, 2.99)	123 (19.8)	0.64 (0.23, 1.81)	117 (18.8)	0.24 (0.09, 0.62)	127 (20.5)	1.71 (0.53, 5.57)
SGA													
Yes	118 (18.0)	21 (17.8)	1.00	21 (17.8)	1.00	30 (25.4)	1.00	25 (21.2)	1.00	28 (23.7)	1.00	29 (24.6)	1.00
No	539 (82.0)	110 (20.4)	1.29 (0.72, 2.34)	111 (20.6)	1.40 (0.76, 2.57)	101 (18.7)	0.67 (0.39, 1.13)	107 (19.9)	1.04 (0.59, 1.85)	103 (19.1)	1.01 (0.57, 1.80)	102 (18.9)	0.76 (0.45, 1.30)
Preterm													
Yes	14 (2.1)	1 (7.1)	1.00	4 (28.6)	1.00	4 (28.6)	1.00	4 (28.6)	1.00	1 (7.1)	1.00	1 (7.1)	1.00
No	643 (97.9)	130 (20.2)	4.63 (0.55, 39.42)	128 (19.9)	0.64 (0.16, 2.57)	127 (19.8)	0.76 (0.19, 2.96)	128 (19.9)	0.42 (0.11, 1.56)	130 (20.2)	4.98 (0.57, 43.66)	130 (20.2)	2.11 (0.26, 16.97)
Toddler nutrition status and primary caregivers at 24 months													
Malnutrition^c^													
Yes	97 (14.8)	27 (27.8)	1.00	24 (24.7)	1.00	23 (23.7)	1.00	21 (21.7)	1.00	20 (20.6)	1.00	25 (25.8)	1.00
No	548 (83.4)	102 (18.6)	0.70 (0.40, 1.21)	106 (19.3)	0.87 (0.49, 1.56)	105 (19.2)	0.92 (0.52, 1.62)	107 (19.5)	1.01 (0.57, 1.80)	111 (20.3)	1.04 (0.58, 1.87)	104 (19.0)	0.72 (0.42, 1.24)
Primary caregivers^d^													
Grandparents	300 (45.7)	53 (17.7)	1.00	60 (20.0)	1.00	55 (18.3)	1.00	66 (22.0)	1.00	53 (17.7)	1.00	65 (21.7)	1.00
Parents	342 (52.1)	76 (22.2)	1.19 (0.79, 1.82)	69 (20.2)	0.96 (0.63, 1.46)	72 (21.1)	1.19 (0.78, 1.80)	63 (18.4)	0.77 (0.51, 1.16)	77 (22.5)	1.53 (1.01, 2.34)	64 (18.7)	0.85 (0.56, 1.28)

OR, odds ratio; CI, confidence interval; MUAC, mid-upper arm circumference; MMN, multiple micronutrient; LBW, low birth weight; SGA, small for gestational age. ^a^MUAC missing 7. ^b^LBW missing 10. ^c^Malnutrition missing 12. ^d^Primary caregivers missing 15.

Children born with a low birth weight (LBW) and preterm showed a marginal association with toddlers’ low levels of concentration (OR = 0.24; 95% CI, 0.09–0.62) and endurance (OR = 0.42; 95% CI, 0.11–1.56), respectively. Malnutrition status in infants was associated with a non-significant increased risk of abnormal activity (OR = 0.70; 95% CI, 0.40–1.21) and motor coordination (OR = 0.72; 95% CI, 0.42–1.24). Having grandparents as the primary caregivers was positively associated with infants’ concentration levels (OR = 1.53; 95% CI, 1.01–2.34) (Table 5).

## Discussion

In this follow-up study, we assessed the behavioral development of toddlers at 24 months of age by the BSID, and mean scores increased for positive behaviors (such as social orientation, cooperativeness, emotional tone, object orientation, endurance, and reactivity) and decreased for negative behaviors (such as fearfulness, tension, and sucking thumb or fingers). Six behavioral factors were identified in children at 24 months of age in rural northwest China: activity, social adaptability, reactivity, endurance, concentration, and motor coordination. Behavioral development interacts with mental and psychomotor development at 24 months. Our findings showed that children with good mental and psychomotor development exhibit behavioral development with better activity, social adaptation ability, reactivity, and endurance. Maternal micronutrient supplementation, household wealth, maternal alcohol exposure and low birth weight were associated with some aspect of behavioral development of infants at 24 months.

### Core behavioral factors of children at 24 months of age

Different ages, environments, and customs of infants present different behavioral features. Core behavioral factors are always present. The main behavioral features of 24-month-old children in rural northwest China are as follows: activity, social adaptability, reactivity, endurance, concentration, and motor coordination. Activity is a key behavioral description and a key aspect of personality at any age^16–18^. Our findings also showed that activity had the greatest impact on the following behavioral rating scales: manipulating, cooperativeness, sucking toys, body motion, energy, and sight. A previous study observed that socially adaptive behavior reflects a child’s language development and perceptual development^19^. Concentration and endurance reflect a child’s self-control ability^19^.

Behavioral problems assessed at age 2 tend to co-occur with delayed motor performance and neurological abnormalities^10,20^. In general, children who have high behavioral scores in physical activity, social adaptiveness, and endurance will have better intellectual and motor development. Our study also showed a positive interaction between mental development and activity, social adaptability, endurance, and concentration, with correlation coefficients ranging from 0.101 to 0.371, and a negative interaction between motor development and concentration with a correlation coefficient of −0.120. Another two studies observed that behavioral problems are strongly related to cognitive impairment, even after adjusting for potential confounding factors^21,22^. Behavioral problems tend to co-occur with lower mental development, which implies that they may share some risk factors.

### The factors associated with behavioral development of children at 24 months of age

Previous studies have observed that maternal alcohol consumption is a risk factor for child behavioral development and could lead to increased levels of behavioral problems^23,24^. Our research showed a similar finding that maternal alcohol consumption could contribute to the decreased activity of children at 24 months of age. Additional environmental risk factor exposure and poor maternal nutrition during pregnancy have consistently been reported as risk factors for cognitive delays and behavioral problems^25–27^. In our study, adverse maternal risk factor exposure and malnutrition appeared to affect infants’ activity development. We also found that maternal micronutrient supplementation positively affected infant behavioral development in social adaptability. In our previous publications, we have reported that prenatal micronutrient supplementation with sufficient iron protects children’s mental development^28^. Because mental development is positively correlated with behavioral development, sufficient maternal micronutrient supplementation could not only improve children’s mental development but also be beneficial to children’s behavioral development.

Some studies have shown an increase in behavioral problems associated with decreased gestational age^10,29^. Children born small for gestational age (SGA) at 2 years corrected age display more anxiety, depression, and withdrawn behaviors^29^. It is well known that gestational age is highly correlated with birth weight^30^. The idea that LBW may increase the risk of behavioral problems is plausible because LBW elevates a child’s risk for inattention^31^. Our results are consistent with the results of reports using the Achenbach’s Child Behavior Checklist and the Teacher’s Report Form in other populations, as well as additional related measures^32^. In addition, there is increasing evidence of a greater risk of behavioral problems among preterm infants that may persist from early childhood into later childhood and adolescence^9,33^. Preterm births coexist with other developmental impairments and are potential markers for later cognitive disorders^33^. Furthermore, we observed that malnourished infants have low activity levels^34,35^. Accumulating evidence suggests that infants who are underweight and stunted exhibit fewer positive effects, lower levels of play, and more insecure attachments^36^. In addition, such children display more attention problems and poorer social relationships, unless the malnutrition can be corrected in a timely fashion^36^.

Many studies also focus on the relationship between children’s growth environment and behavioral problems^37,38^. Publications have suggested that children from low-income households are approximately twice as likely as children from high-income households to display behavioral problems at 24 months of age^8^. Our study also showed that a higher economic status positively affected infants’ behavioral development in social adaptability and endurance. In addition, children demonstrated better concentration when their primary caregivers were grandparents, which was largely attributable to fewer constraints and more patience with playing and learning from their keepers^39^. However, another study in an urban area in China showed that children who were brought up by grandparents had more behavioral problems than children reared by their parents^40^. Evidence suggests that maternal sensitivity and responsivity are associated with higher infant cognitive ability and fewer behavioral problems in preschool children^41,42^. Furthermore, high-quality parenting may help reduce the negative effects on a child’s behavioral development^43^.

The period of 0 to 5 years is a crucial time during which the majority of the neuropsychological, socio-emotional, and behavioral competencies of the child develop, influencing one another and reaching a high level of integration^16^. Moreover, 24 months of age is a key time in infant development^6^. Behavioral problems are potentially modifiable^44–46^, and the period from 0 to 2 years of age offers a unique opportunity for intervention because the child’s personality is not yet fully structured and the possibilities of achieving a change are greater than later in childhood. Recent randomized controlled trials have demonstrated that the behaviors and social skills of children born with adverse outcomes from pregnancy can be improved by specific intervention programs^47–49^. It may therefore be important to identify children that are affected by adverse pregnancy outcomes early and try to prevent these problems through appropriate intervention programs.

Admittedly, our research also has some limitations. First of all, the assessment tool used in this study was the Bayley Scale II-Chinese Version, which was developed based on children living in urban areas. All the children involved in this study lived in rural areas, which were characterized by prominent public problems with maternal and child malnutrition. Therefore, the mean MDI score of children in this study was significant lower than the scores of children in an urban area. Secondly, the children in this study may have certain particularities because their mother enrolled in a prenatal micronutrient supplementation trial, so the generalization of the findings should be done with caution. Thirdly, although the standardized scores of behavioral factors obtained through factor analysis are commonly used in the analysis of behavioral features, they are only applicable to the comparison of subgroups within the study population to find out the factors associated with behavioral development. The direct comparison of behavioral factor scores with the results from other similar studies are irrational and limited.

Children’s behavioral development is a long-term and complex process that is influenced by many factors. Comprehensive interventions, including maternal micronutrient supplementation, controlling maternal alcohol exposure and reducing the incidence of low birth weight, may have potential effects on improving the behavioral development of children at 24 months in northwestern China. Larger population-based studies with more appropriate assessment tools are needed to verify the effectiveness of these interventions.

## Materials and Methods

### Study design and population

This study focused on the follow-up assessments of the mental, psychomotor and behavioral development of children at 24 months of age. These children were born to women recruited in the cluster randomized, double-blinded, controlled trial of prenatal micronutrient supplementation with three treatment arms (MMN, iron/folic acid, and folic acid) conducted in two rural counties in northwest China from 2002 to 2006. The primary objective of the main trial was to evaluate the impact of micronutrient supplements during pregnancy on birth weight, duration of gestation at delivery, and perinatal mortality, and the details of this trial have been described elsewhere^50^. Briefly, the pregnant women in the designated villages were randomly assigned to 3 treatment arms: folic acid (400 µg folic acid), iron-folic acid (60 mg iron and 400 µg folic acid), or MMN (30 mg iron, 400 µg folate, 15 mg zinc, 2 mg copper, 65 µg selenium, 150 µg iodine, 800 µg vitamin A, 1.4 mg vitamin B1, 1.4 mg vitamin B2, 1.9 mg vitamin B6, 2.6 µg vitamin B12, 5 µg vitamin D, 70 mg vitamin C, 10 mg vitamin E, and 18 mg niacin). The three supplement tablets were indistinguishable from one another in both appearance and taste and were packaged in identical blister packs. None of the investigators or participants knew the treatment distribution until the original trial was completed. All the protocols were approved by the Committee for Science and Research at Xi’an Jiaotong University.

### Eligibility for follow-up assessment

Women residing in the study area who were less than 28 gestational weeks pregnant during the recruitment period and consented to participate in the trial were included. The baseline information for newly identified pregnant women was collected by an initial antenatal care check, and a blister pack containing 15 tablets with instructions to take one tablet daily was provided. The village doctor visited the women twice each month to retrieve the used blister packs and to deliver a new blister pack containing 15 additional capsules. The birth outcomes, including birth weight, birth length, Apgar scores at 5 minutes and other birth details, were collected by midwives for hospital deliveries or by village doctors for home deliveries^48^. Altogether, 5828 eligible women were recruited for the trial, and 4697 live births were recorded. In this study, a total of 1303 singleton live births between May 2004 and January 2006 met the inclusion criteria of the follow-up study and were accompanied by parental consent. Infants who had an obvious deformity or other birth defects and infants who could not complete the questionnaire survey or physical examination were excluded. Among those children, 157 (12.0%) missed their development assessment at 24 months of age and another 489 (37.5%) completed only the Mental Development and Psychomotor Development Assessment. A total of 657 (50.4%) infants completed the BSID assessment. The study size was calculated to detect a difference of 0.1 standard deviation in the BSID’s MDI at 24 months corrected age with a power of 80% using a two-sided test (α = 0.05). Sample size calculations indicated that a minimum of 400 infants was required.

### Follow-up and data collection

From May 2004 to May 2008, a follow-up study was conducted to assess the development of these children using the BSID II-Chinese Version, including the mental scale, the psychomotor scale and IBR, which were translated into Chinese and locally standardized to assure cultural appropriateness. The reliability and validity of these standardized scales have been shown to be satisfactory^51^.

The mental scale in the BSID measures the child’s performance on tasks requiring memory, problem solving, and language skills. The psychomotor scale in the BSID measures a child’s gross and fine motor skills, such as his or her ability to grasp, stand, walk, run, and write. The mental development raw score and psychomotor development raw score signify the items that a child passes on the mental and psychomotor scales of the BSID, respectively. The MDI and PDI are nonlinear transformations of the raw scores, using standard procedures that are based on data for Chinese children^52,53^.

The IBR reflects an infant’s orientation to objects and people, emotional state, and developmental level. The IBR scale comprises 30 items, 25 of which are five-point (1 to 5 scores) or six-point (1 to 6 scores) rating scales with a score of 1 representing ‘consistently lacking in this behavior performance’ and a score of 5 or 6 representing ‘consistently good in this behavior performance’.

The BSID assessment and anthropometry of each child were collected at 24 months of age (±15 days) after written informed consent was obtained from the guardians. The BSID assessments were administered by specially trained examiners, who had demonstrated high levels of consistency with one another, at the village clinic or in the child’s home in a standardized manner, when the child was not hungry, sleepy, or restless. Another assessment was arranged at a later date if the child was sick, unavailable, or would not cooperate. The examiners and the participating women were blinded to the supplementation assignments. Body weight was measured with an electronic scale with precision to the nearest 100 grams (type HD 305, Tanita, Dongguan, Guangdong Province, China). A portable measuring board measured body length to the nearest 1 millimeter.

Data collection of baseline information and birth outcomes was performed as described in previously published papers regarding this trial^54^.

### Definitions of risk factors associated with infant behavioral development

The primary objective of this study was to explore the risk factors associated with infant behavioral development for intervention development; therefore, the analysis focused on identifying the potential modifiable risk factors in maternal nutrition status, maternal environmental risk factor exposure, adverse birth outcomes, nutrition status and primary caregivers during infancy.

Maternal malnutrition was defined as mid-upper arm circumference (MUAC) ≤ 23.5 cm, which was determined at the baseline interview^19^. Maternal smoke exposure was defined as active smoking (smoking more than one cigarette per day) or passive smoking (family members smoking cigarettes in the presence of the pregnant woman). Maternal alcohol exposure was defined as drinking Chinese wine more than once per week. Toxic chemical exposure was defined as women being frequently exposed to pesticides or other toxic chemicals during pregnancy.

LBW, preterm delivery and SGA were considered common adverse birth outcomes^9^. The gestational age at birth was estimated by the mother’s last menstrual period; we used completed days based on the first day of the last menstrual period. Preterm delivery was defined as a delivery with a gestational age of less than 37 completed weeks. LBW was defined as a birth weight < 2500 g. SGA babies were defined as having a birth weight below the 10th percentile of weight-for-age and sex, as defined by the Intergrowth standards^55^.

The Z-score method was used to evaluate the nutritional status of infants, which was calculated according to the 2006 WHO child growth standards^56^. Malnutrition was defined as situations in which height-for-age, weight-for-age, or weight-for-height were less than the international reference value by more than two standard deviations. A household wealth index was constructed from an inventory of 16 household assets or facilities by using principal components analysis^50^, and household wealth was categorized into tertiles indicating the poorest, middle, and wealthiest households.

### Data analysis

All data were checked manually for completeness and were double-entered into a data management system. To avoid any inconsistencies, we subjected the data to range checks and logical checks for accuracy.

The background characteristics of the families and infants were described by mean and standard deviations for continuous variables or frequency and percentages for categorical variables. Mean, standard deviation, median and quartiles were provided for MDI, PDI, and behavioral scores. An exploratory factor analysis of 25 rating scales from Bayley’s IBR was conducted to extract the major behavioral factors and the standardized scores (with a mean of 0 and a standard deviation of 1) for each behavioral factor that was calculated; eigenvalues > 1.0 was used as the retained criterion and the method of varimax rotation was used to render those factors clearer and more definite. Spearman and partial correlations were used to determine associations between infant behavior scores and mental and psychomotor development. We considered the following variables as potential confounders in the partial correlation analysis: infant’s gender, birth weight, gestational weeks at delivery, Apgar scores at 5 minutes, age of toddler at assessment, mother’s age at delivery, MUAC at enrollment, micronutrient supplementation during pregnancy, number of supplement tablets consumed, maternal education level and occupation, and paternal education level and occupation.

The standardized scores of behavioral factors were categorized into quintiles; we defined the lowest quintile as abnormal infant behavioral development. A multivariable logistic regression model was then used to explore the associated factors of infant behavioral development, including maternal malnutrition; exposure to smoke, alcohol, or toxic chemicals during pregnancy; prenatal factors; early nutrition status; and primary caregivers during infancy.

All analyses were conducted with SPSS 18.0 (SPSS, Chicago, IL). Additionally, all reported *P* values were two-tailed, and a difference of *P* < 0.05 was considered significant. All methods were performed in accordance with the relevant guidelines and regulations.

### Ethics approval

Our research in two rural counties of northwest China was approved by the Ethical Committee of the College of Medicine, Xi’an Jiaotong University (NO: 2013–093). Written informed consent was obtained from the guardians of every investigated child aged 6–36 months old.

## Electronic supplementary material

Supplementary Table S1; Supplementary Table S2

## Data Availability

All data generated or analyzed during this study are included in this published article (and its Supplementary Information files).
