# Supplementary material for: Features and Associated Factors of the Behavioral Development of 24-month-old Children in Rural China: Follow-up Evaluation of a Randomized Controlled Trial
Source: Sci Rep. 2018 Sep 18;8:13977. doi: 10.1038/s41598-018-32171-1 (PMC6143539; doi:10.1038/s41598-018-32171-1)
Supplement: Supplementary file 1 — Supplementary Table S1; Supplementary Table S2 [file 41598_2018_32171_MOESM1_ESM.pdf]

Features and Associated Factors of the Behavioral Development of 24-month-old Children in Rural China: Follow-up Evaluation of a Randomized Controlled Trial

Xue Yang, Zhaoyang Yin, Yue Cheng, Wenfang Yang, Zhonghai Zhu, Min Zhang, Danyang Li, Danli Liu, Hong Yan & Lingxia Zeng*

Supplementary Table S1 Comparison of baseline characteristics in different BSID completed groups

Characteristic	Completed BSID		*P*
	Yes(n=657)	No(n=646)	
Demographic characteristics			
Maternal educational level, n (%)			0.098
Primary school or less	175 (26.6)	207 (32.4)	
Secondary school	396 (60.3)	363 (56.2)	
High school or above	86 (13.1)	76 (11.8)	
Maternal occupation, n (%)			0.680
Farmer	555 (84.5)	551 (85.3)	
Other	102 (15.5)	95 (14.7)	
Paternal educational level, n (%) [a]			0.203
Primary school or less	65 (9.9)	74 (11.5)	
Secondary school	449 (68.5)	455 (70.8)	
High school or above	140 (21.4)	114 (17.7)	
Paternal occupation, n (%)			0.287
Farmer	492 (74.9)	500 (77.4)	
Other	165 (25.1)	146 (22.6)	
Household wealth during pregnancy, n (%)			0.120
Poor	138 (21.0)	166 (25.7)	
Middle	271 (41.3)	258 (39.9)	
Wealthy	248 (37.8)	222 (34.4)	
Maternal nutritional status and micronutrient supplement during pregnancy			
Age at delivery,mean±SD,y	25.5±4.6	25.3±0.4	0.147
MUAC at enrollment,mean±SD,cm	23.1±1.8	23.2±1.8	0.141
Micronutrient supplement, n (%)			0.948
Folic acid	236 (35.9)	230 (35.6)	
Iron-folic acid	223 (33.9)	216 (33.4)	
MMN	198 (30.1)	200 (30.9)	
No.of supplement tablets,mean±SD	173.3±41.1	167.4±41.7	0.011

Birth outcomes			
Gestation at birth,mean±SD,w	39.9±1.6	39.9±1.5	0.383
Birth weight,mean±SD,g	3179.5±409.0	3189.1±411.0	0.672
Birth length, mean±SD, cm	49.2±2.5	49.0±2.5	0.142
Birth head circumference, mean±SD, cm	33.2±1.6	33.4±1.4	0.086
Apgar score at 5min after birth,mean±SD	9.9±0.5	9.8±0.6	0.025
Gender,n (%)			0.853
Boy	404 (61.5)	394 (61.0)	
Girl	253 (38.5)	252 (39.0)	
Toddler characteristics at 24 months			
Age of the assessment,mean±SD,d	24.5±0.6	24.3±0.41	<0.001
Length, mean±SD, cm	84.6±3.2	84.6±3.4	0.853
Weight, mean±SD, kg	11.7±1.3	11.5±1.2	0.065
Primary caregivers, n (%) [b]			0.078
Parents	342 (53.3)	245 (48.0)	
Grandparents	300 (46.7)	265 (52.0)	

BSID, Bayley scale of infant development; SD, standard deviation; MUAC, mid-upper arm circumference; MMN, multiple micronutrient. [a]Paternal educational level missing 6. [b]Primary caregivers missing 15.

Supplementary Table S2 Univariate analysis of factors associated with infant behavioral development at 24 months of age

Index	N (%)	Activity		Social adaptability		Reactivity		Endurance		Concentration		Motor coordination	
		mean±SD	*P*	mean±SD	*P*	mean±SD	*P*	mean±SD	*P*	mean±SD	*P*	mean±SD	*P*
Maternal nutrition status and adverse exposure during pregnancy													
MUAC [a]			0.164		0.984		0.972		0.527		0.982		0.726
≤23.5	405 (61.6)	-0.040±1.037		0.003±0.998		-0.001±1.012		-0.020±0.995		-0.002±1.004		-0.012±1.012	
＞23.5	245 (37.3)	0.073±0.941		0.004±1.024		0.001±0.985		0.031±0.994		-0.001±1.003		0.017±0.988	
Micronutrient supplementation			0.381		0.195		0.500		0.370		0.994		0.291
Folic acid	236 (35.9)	-0.025±1.011		-0.076±1.033		0.036±1.046		-0.034±1.081		-0.004±0.946		0.050±0.937	
Iron –folic acid	223 (33.9)	-0.046±1.036		-0.006±0.989		-0.064±0.969		0.077±0.910		-0.002±1.033		0.029±1.050	
MMN	198 (30.1)	0.081±0.944		0.098±0.969		0.029±0.979		-0.046±0.997		0.006±1.030		-0.092±1.014	
No. of supplement tablets			0.579		0.198		0.392		0.951		0.948		0.839
≤180	325 (49.5)	-0.022±1.024		-0.051±1.033		-0.034±1.030		0.002±1.053		0.003±0.985		0.008±1.020	
＞180	332 (50.5)	0.021±0.977		0.050±0.966		0.033±0.970		-0.002±0.946		-0.003±1.016		-0.008±0.981	
Smoke exposure			0.434		0.466		0.665		0.331		0.398		0.408
Yes	368 (56.0)	-0.027±0.982		0.025±0.971		0.015±0.975		-0.034±1.001		0.030±0.920		0.029±1.029	
No	289 (44.0)	0.034±1.023		-0.032±1.036		-0.019±1.032		0.043±0.998		-0.038±1.094		-0.036±0.963	
Toxic chemical exposure			0.004		0.998		0.184		0.078		0.448		0.365
Yes	153 (23.3)	-0.195±1.047		-0.009±1.016		-0.088±0.985		-0.124±0.948		0.045±0.992		-0.066±1.037	
No	504 (76.7)	0.071±0.973		-0.009±0.995		0.035±1.010		0.039±1.015		-0.025±1.001		0.018±0.991	
Alcohol exposure			0.034		0.057		0.512		0.340		0.530		0.320
Yes	18 (2.7)	-0.490±1.120		0.444±0.821		-0.147±0.904		0.222±0.918		0.145±0.972		0.238±0.906	
No	626 (95.3)	0.019±1.000		-0.008±0.997		0.010±1.005		-0.007±1.006		-0.006±1.002		0.002±0.997	
Household wealth during pregnancy			0.019		0.001		0.181		<0.001		0.500		0.590
Poor	138 (21.0)	-0.013±0.978		-0.214±1.116		-0.131±1.082		-0.265±1.047		0.063±0.907		-0.077±0.981	

Middle	271 (41.3)	-0.114±0.996		-0.042±0.943		0.008±0.966		-0.006±0.940		0.018±1.019		0.027±1.031	
Wealthy	248 (37.8)	0.132±1.004		0.165±0.968		0.064±0.987		0.154±1.009		-0.055±1.028		0.013±0.977	
Adverse birth outcomes													
LBW [b]			0.453		0.965		0.446		0.773		0.117		0.934
Yes	26 (4.0)	-0.135±0.977		-0.003±0.801		-0.147±1.182		-0.055±1.219		-0.409±1.321		-0.025±0.737	
No	621 (94.5)	0.014±0.996		0.004±1.011		0.005±0.989		0.003±0.995		0.015±0.981		-0.008±1.013	
SGA			0.309		0.748		0.223		0.136		0.887		0.031
Yes	118 (18.0)	0.085±1.023		0.027±0.992		-0.110±1.103		0.124±1.018		-0.012±1.104		-0.180±0.998	
No	539 (82.0)	-0.019±0.995		-0.006±1.003		0.024±0.975		-0.027±0.995		0.003±0.977		0.039±0.997	
Preterm			0.688		0.369		0.378		0.061		0.153		0.254
Yes	14 (2.1)	0.106±0.796		0.238±1.234		-0.246±1.023		-0.639±1.188		0.378±1.082		0.302±0.688	
No	643 (97.9)	-0.002±1.004		-0.005±0.995		0.005±1.000		0.014±0.992		-0.008±0.977		-0.007±1.005	
Toddler nutrition status and primary caregivers at 24 months													
Malnutrition [c]			0.003		0.434		0.112		0.201		0.888		0.243
Yes	97 (14.8)	-0.315±1.144		-0.076±1.041		-0.146±1.023		-0.110±1.020		0.010±0.970		-0.110±1.100	
No	548 (83.4)	0.058±0.968		0.010±0.988		0.029±0.993		0.030±1.000		-0.010±1.010		0.020±0.990	
Primary caregivers [d]			0.046		0.118		0.314		0.859		0.962		0.167
Grandparents	300 (45.7)	0.089±1.009		-0.070±0.964		0.043±0.998		0.120±1.109		-0.002±0.960		-0.064±1.032	
Parents	342 (52.1)	-0.070±0.999		0.053±1.017		-0.036±1.003		-0.002±0.905		-0.005±1.044		0.046±0.978	

SD, standard deviation; MUAC, mid-upper arm circumference; MMN, multiple micronutrient; LBW, low birth weight; SGA, small for gestational age. [a]MUAC missing 7. [b]LBW missing 10. [c]Malnutrition missing 12. [d]Primary caregivers missing 15.
